# Supplementary material for: Frequency of Common and Uncommon BRAF Alterations among Colorectal and Non-Colorectal Gastrointestinal Malignancies
Source: Cancers (Basel). 2024 May 10;16(10):1823. doi: 10.3390/cancers16101823 (PMC11119877; doi:10.3390/cancers16101823)
Supplement: Supplementary file 1 [file cancers-16-01823-s001.zip › cancers-2942372-supplementary.pdf]

Supplemental Table 1: Description of cohort and incidence of *BRAF* gain-of-function mutation by primary tumor location (detailed).

**Characteristic**	**Overall**, N = 51,560[1]	**BRAF_wt**, N = 49,044[2]	**BRAF_GOF**, N = 2,516[2]
Colon	14,350 (28%)	12,794 (89%)	1,556 (11%)
Pancreas	14,300 (28%)	14,041 (98%)	259 (1.8%)
Rectum	5,479 (11%)	5,235 (96%)	244 (4.5%)
Esophagus	3,316 (6.4%)	3,286 (99%)	30 (0.9%)
Stomach	3,029 (5.9%)	2,981 (98%)	48 (1.6%)
Intrahepatic bile duct	2,370 (4.6%)	2,244 (95%)	126 (5.3%)
Liver	1,585 (3.1%)	1,566 (99%)	19 (1.2%)
Gallbladder	1,023 (2.0%)	1,004 (98%)	19 (1.9%)
Rectosigmoid junction	827 (1.6%)	789 (95%)	38 (4.6%)
Biliary tract	792 (1.5%)	763 (96%)	29 (3.7%)
Appendix	788 (1.5%)	770 (98%)	18 (2.3%)
Extrahepatic bile duct	738 (1.4%)	712 (96%)	26 (3.5%)
Gastrointestinal tract	665 (1.3%)	636 (96%)	29 (4.4%)
Anus	553 (1.1%)	545 (99%)	8 (1.4%)
Ampulla of Vater	508 (1.0%)	485 (95%)	23 (4.5%)
Small intestine	489 (0.9%)	475 (97%)	14 (2.9%)
Duodenum	414 (0.8%)	395 (95%)	19 (4.6%)
Intestinal tract	144 (0.3%)	144 (100%)	0 (0%)
Jejunum	104 (0.2%)	94 (90%)	10 (9.6%)
Ileum	86 (0.2%)	85 (99%)	1 (1.2%)

[1] n (%), note that percentage indicates column percent

[2] n (%), note that percentage indicates row percent

Supplemental Table 2: Frequency of *BRAF* gain-of-function alteration by primary cancer location

**Characteristic**	**Overall**, N = 2,516[1]	**CRC**, N = 1,838[1]	**Other GI**, N = 678[1]	**p-value**[2]

**BRAF alteration class**				<0.001
SV	2,367 (94%)	1,791 (97%)	576 (85%)	
Fusion	116 (4.6%)	35 (1.9%)	81 (12%)	
CNA	25 (1.0%)	6 (0.3%)	19 (2.8%)	
SV, Fusion	6 (0.2%)	6 (0.3%)	0 (0%)	
CNA, Fusion	1 (<0.1%)	0 (0%)	1 (0.1%)	
SV, CNA	1 (<0.1%)	0 (0%)	1 (0.1%)	
**Short variant**	2,374 (94%)	1,797 (98%)	577 (85%)	<0.001
**Copy number amplificatio n**	27 (1.1%)	6 (0.3%)	21 (3.1%)	<0.001
**Fusion**	123 (4.9%)	41 (2.2%)	82 (12%)	<0.001

[1]n(%)

[2]Fisher's exact test; Pearson's Chi-squared test

Supplemental Table 3: *BRAF* fusion by patient, orientation agnostically listed

Fusion Gene	Overall, N = 2,516	CRC, N = 1,838	Other GI, N = 678
BRAF-SND1	31 (1.2%)	3 (0.2%)	28 (4.1%)
BRAF-TRIM24	11 (0.4%)	5 (0.3%)	6 (0.9%)
BRAF-MKRN1	7 (0.3%)	4 (0.2%)	3 (0.4%)
BRAF-CCNY	3 (0.1%)	0 (0%)	3 (0.4%)

BRAF-RP1-167F1.2	3 (0.1%)	2 (0.1%)	1 (0.1%)
AGAP3-BRAF	2 (<0.1%)	1 (<0.1%)	1 (0.1%)
AKAP9-BRAF	2 (<0.1%)	1 (<0.1%)	1 (0.1%)
BRAF-COBLL1	2 (<0.1%)	0 (0%)	2 (0.3%)
BRAF-GTF2IRD1	2 (<0.1%)	2 (0.1%)	0 (0%)
BRAF-NRF1	2 (<0.1%)	1 (<0.1%)	1 (0.1%)
BRAF-ZC3HAV1	2 (<0.1%)	1 (<0.1%)	1 (0.1%)
AC073934.6-BRAF	1 (<0.1%)	0 (0%)	1 (0.1%)
AGAP1-BRAF	1 (<0.1%)	0 (0%)	1 (0.1%)
ANK3-BRAF	1 (<0.1%)	0 (0%)	1 (0.1%)
ATP11B-BRAF	1 (<0.1%)	1 (<0.1%)	0 (0%)
BAIAP2L1-BRAF	1 (<0.1%)	1 (<0.1%)	0 (0%)
BRAF-CCDC186	1 (<0.1%)	0 (0%)	1 (0.1%)
BRAF-CEP290	1 (<0.1%)	0 (0%)	1 (0.1%)
BRAF-CERT1	1 (<0.1%)	0 (0%)	1 (0.1%)
BRAF-CHCHD3	1 (<0.1%)	1 (<0.1%)	0 (0%)
BRAF-COG3, BRAF-PHF2P2	1 (<0.1%)	0 (0%)	1 (0.1%)
BRAF-COG5	1 (<0.1%)	0 (0%)	1 (0.1%)
BRAF-CUX1	1 (<0.1%)	0 (0%)	1 (0.1%)
BRAF-DENND2A	1 (<0.1%)	1 (<0.1%)	0 (0%)
BRAF-DENND2A, BRAF-POLR1D	1 (<0.1%)	0 (0%)	1 (0.1%)
BRAF-ERC1	1 (<0.1%)	0 (0%)	1 (0.1%)
BRAF-ESRP1	1 (<0.1%)	0 (0%)	1 (0.1%)
BRAF-EXOC4	1 (<0.1%)	0 (0%)	1 (0.1%)
BRAF-GOLGB1	1 (<0.1%)	0 (0%)	1 (0.1%)
BRAF-KDM7A	1 (<0.1%)	1 (<0.1%)	0 (0%)
BRAF-KIAA0754, BRAF-MACF1	1 (<0.1%)	1 (<0.1%)	0 (0%)
BRAF-KIAA1217	1 (<0.1%)	0 (0%)	1 (0.1%)
BRAF-KTN1	1 (<0.1%)	0 (0%)	1 (0.1%)
BRAF-LINC00882	1 (<0.1%)	1 (<0.1%)	0 (0%)
BRAF-MCC	1 (<0.1%)	0 (0%)	1 (0.1%)

BRAF-MGAM	1 (<0.1%)	0 (0%)	1 (0.1%)
BRAF-MKLN1	1 (<0.1%)	1 (<0.1%)	0 (0%)
BRAF-PRSS1	1 (<0.1%)	0 (0%)	1 (0.1%)
BRAF-PTPN1	1 (<0.1%)	1 (<0.1%)	0 (0%)
BRAF-PTPN12	1 (<0.1%)	1 (<0.1%)	0 (0%)
BRAF-PTPRN2	1 (<0.1%)	0 (0%)	1 (0.1%)
BRAF-RAVER2	1 (<0.1%)	1 (<0.1%)	0 (0%)
BRAF-RP11-689K5.3, BRAF-SEC31A	1 (<0.1%)	0 (0%)	1 (0.1%)
BRAF-RPL12	1 (<0.1%)	1 (<0.1%)	0 (0%)
BRAF-RPS25	1 (<0.1%)	0 (0%)	1 (0.1%)
BRAF-RRBP1	1 (<0.1%)	0 (0%)	1 (0.1%)
BRAF-SEC24D	1 (<0.1%)	0 (0%)	1 (0.1%)
BRAF-SGK3	1 (<0.1%)	0 (0%)	1 (0.1%)
BRAF-SLC37A3	1 (<0.1%)	0 (0%)	1 (0.1%)
BRAF-SLC4A4	1 (<0.1%)	0 (0%)	1 (0.1%)
BRAF-SND1, BRAF-TBXAS1	1 (<0.1%)	0 (0%)	1 (0.1%)
BRAF-SPAG16	1 (<0.1%)	0 (0%)	1 (0.1%)
BRAF-SPECC1L	1 (<0.1%)	0 (0%)	1 (0.1%)
BRAF-STK39	1 (<0.1%)	0 (0%)	1 (0.1%)
BRAF-STRN3	1 (<0.1%)	1 (<0.1%)	0 (0%)
BRAF-TMEM178B	1 (<0.1%)	0 (0%)	1 (0.1%)
BRAF-TMEM44	1 (<0.1%)	1 (<0.1%)	0 (0%)
BRAF-TNS3	1 (<0.1%)	1 (<0.1%)	0 (0%)
BRAF-TRIM44	1 (<0.1%)	0 (0%)	1 (0.1%)
BRAF-ZKSCAN5	1 (<0.1%)	0 (0%)	1 (0.1%)
CN amp, BRAF-DENND11, BRAF-HIPK2	1 (<0.1%)	0 (0%)	1 (0.1%)
p.Asp594Gly, BRAF-MKRN1, BRAF-TRIM24	1 (<0.1%)	1 (<0.1%)	0 (0%)
p.Gly469Ala, p.Gly466Ala, BRAF-NDUFB2	1 (<0.1%)	1 (<0.1%)	0 (0%)
p.Val600Glu, ATR-BRAF, BRAF-MIGA2	1 (<0.1%)	1 (<0.1%)	0 (0%)

p.Val600Glu, BRAF-TRIM24	1 (<0.1%)	1 (<0.1%)	0 (0%)
p.Val600Glu, BRAF-UBE3C	1 (<0.1%)	1 (<0.1%)	0 (0%)
p.Val600Glu, BRAF-ZW10	1 (<0.1%)	1 (<0.1%)	0 (0%)

Supplemental Table 4: Microsatellite instability and tumor mutation burden among patients with *BRAF* gain-of-function mutation by primary cancer location.

	**Overall**, N = 2,516[1]	**CRC**, N = 1,838[1]	**Other GI**, N = 678[1]	**p-value**[2]
**TMB (mut/Mb)**				<0.001
Not TMB-high	1,409 (75%)	940 (69%)	469 (93%)	
TMB-high	459 (25%)	425 (31%)	34 (6.8%)	
Unknown	648	473	175	
**MSI**				<0.001
Not detected/Low/Stable	2,007 (80%)	1,358 (74%)	649 (96%)	
High	505 (20%)	476 (26%)	29 (4.3%)	
Unknown	4	4	0	

* TMB-high is defined as ≥10 mutations/megabase in tissue and ≥15 mutations/megabase in blood.

[1] n (%)

[2] Pearson's Chi-squared test

Supplemental Table 5. Prevalence of co-mutations in colorectal cancers and non-colorectal GI cancers with *BRAF* alterations.

	**CRC**, N = 1,838[1]	**Other GI**, N = 678[1]	**p-value**[2]	**q-value**[3]
**TP53**	1,295 (70%)	350 (52%)	<0.001	<0.001
**APC**	781 (42%)	85 (13%)	<0.001	<0.001
**CDKN2A**	96 (5.2%)	220 (32%)	<0.001	<0.001
**RNF43**	548 (30%)	35 (5.2%)	<0.001	<0.001
**SMAD4**	410 (22%)	117 (17%)	0.006	0.008
**PIK3CA**	389 (21%)	67 (9.9%)	<0.001	<0.001
**CDKN2B**	73 (4.0%)	137 (20%)	<0.001	<0.001
**MSH3**	337 (18%)	21 (3.1%)	<0.001	<0.001
**KMT2D**	324 (18%)	40 (5.9%)	<0.001	<0.001

**ARID1A**	317 (17%)	114 (17%)	0.8	0.8
**PTEN**	305 (17%)	27 (4.0%)	<0.001	<0.001
**KRAS**	212 (12%)	112 (17%)	<0.001	0.001
**FBXW7**	294 (16%)	25 (3.7%)	<0.001	<0.001
**MTAP**	36 (2.0%)	106 (16%)	<0.001	<0.001
**ALK**	271 (15%)	103 (15%)	0.8	0.8
**MSH6**	233 (13%)	16 (2.4%)	<0.001	<0.001
**RET**	210 (11%)	85 (13%)	0.4	0.5
**ACVR2A**	228 (12%)	20 (2.9%)	<0.001	<0.001
**UGT1A1**	156 (8.5%)	78 (12%)	0.021	0.026
**KMT2C**	195 (11%)	33 (4.9%)	<0.001	<0.001
**BRCA2**	192 (10%)	49 (7.2%)	0.015	0.02
**NTRK3**	132 (7.2%)	70 (10%)	0.01	0.013
**NOTCH1**	189 (10%)	43 (6.3%)	0.002	0.003
**ZNRF3**	182 (9.9%)	16 (2.4%)	<0.001	<0.001
**LRP1B**	180 (9.8%)	33 (4.9%)	<0.001	<0.001
**RAD50**	177 (9.6%)	11 (1.6%)	<0.001	<0.001
**ASXL1**	173 (9.4%)	15 (2.2%)	<0.001	<0.001
**HNF1A**	159 (8.7%)	8 (1.2%)	<0.001	<0.001
**B2M**	147 (8.0%)	11 (1.6%)	<0.001	<0.001

**NRG1**	146 (7.9%)	49 (7.2%)	0.6	0.6
**ATM**	145 (7.9%)	40 (5.9%)	0.09	0.11
**BCOR**	144 (7.8%)	25 (3.7%)	<0.001	<0.001
**CREBBP**	141 (7.7%)	8 (1.2%)	<0.001	<0.001
**FLCN**	141 (7.7%)	13 (1.9%)	<0.001	<0.001
**BCL6**	140 (7.6%)	45 (6.6%)	0.4	0.4
**CYP1B1**	132 (7.2%)	41 (6.0%)	0.3	0.4
**TCF7L2**	132 (7.2%)	6 (0.9%)	<0.001	<0.001
**NTRK2**	52 (2.8%)	46 (6.8%)	<0.001	<0.001
**CIC**	123 (6.7%)	17 (2.5%)	<0.001	<0.001
**RPL22**	122 (6.6%)	9 (1.3%)	<0.001	<0.001
**BCORL1**	120 (6.5%)	14 (2.1%)	<0.001	<0.001
**CASP8**	115 (6.3%)	7 (1.0%)	<0.001	<0.001
**PTCH1**	115 (6.3%)	7 (1.0%)	<0.001	<0.001
**ARID1B**	114 (6.2%)	20 (2.9%)	0.001	0.002
**PIK3R1**	111 (6.0%)	14 (2.1%)	<0.001	<0.001
**TERT**	40 (2.2%)	40 (5.9%)	<0.001	<0.001
**ZFHX3**	107 (5.8%)	13 (1.9%)	<0.001	<0.001
**ARID2**	95 (5.2%)	38 (5.6%)	0.7	0.7

**MTAP-downstream**	9 (0.5%)	37 (5.5%)	<0.001	<0.001
**NF1**	100 (5.4%)	29 (4.3%)	0.2	0.3
**FAT1**	99 (5.4%)	17 (2.5%)	0.002	0.003
**PMS2**	95 (5.2%)	28 (4.1%)	0.3	0.3
**GNAS**	85 (4.6%)	35 (5.2%)	0.6	0.6
**ATR**	94 (5.1%)	7 (1.0%)	<0.001	<0.001
**CHD2**	94 (5.1%)	4 (0.6%)	<0.001	<0.001
**MLH3**	93 (5.1%)	4 (0.6%)	<0.001	<0.001
**FGFR3**	69 (3.8%)	34 (5.0%)	0.2	0.2
**MYB**	56 (3.0%)	34 (5.0%)	0.018	0.024
**AMER1**	92 (5.0%)	12 (1.8%)	<0.001	<0.001

* Somatic alterations include short variant pathogenic/likely pathogenic mutations and copy number alterations (loss or amplification, copy number 0 or ≥8, respectively)

[1] n (%)

[2] Pearson's Chi-squared test

[3] False discovery rate correction for multiple testing
